# A Treatise on Human Physiology

**Published:** 1867-10

**Authors:** 


A Treatise on Human Physiology; Designed for the Use of Students and Practitioners of Medicine. By JOHN C. DALTON, M.D., Prof. of Physiology and Microscopic Anatomy in the College of Physicians and Surgeons, New York; Member of the New York Academy of Medicine, etc., etc., etc. Fourth Edition, revised and enlarged. With 274 Illustrations. Philadelphia: HENRY C. LEA. 1867.

This is a new edition of Dr. Dalton's well-known work on Human Physiology. It is only necessary to say that it is published in excellent style, and constitutes one of the best text-books on physiology that we have in the English language.

For sale by S. C. GRIGGS & Co., 39 and 41 Lake St., Chicago. Price $5.25.

